# Secreted and Transmembrane 1A Is a Novel Co-Stimulatory Ligand

**DOI:** 10.1371/journal.pone.0073610

**Published:** 2013-09-10

**Authors:** Duncan Howie, Hugo Garcia Rueda, Marion H. Brown, Herman Waldmann

**Affiliations:** Sir William Dunn School of Pathology, University of Oxford, Oxford, United Kingdom; University of Miami, United States of America

## Abstract

Most T cell responses to pathogens or self antigens are modulated through the action of regulatory T cells and tissue-specific inhibitory mechanisms. To this end, several receptor-ligand pairs have evolved which either augment or diminish T cell function. Here we describe the tissue ligand SECTM1A (Secreted and transmembrane1A) as an alternative murine CD7 ligand. We show that SECTM1A, like SECTM1B, binds strongly to CD7, and that SECTM1B was able to compete with SECTM1A for CD7 binding. SECTM1A is ubiquitously expressed and has two major alternative transcripts which differ in expression between tissues. Both immobilised soluble forms of SECTM1A and SECTM1B and cell surface anchored forms demonstrated opposing effects on CD4+ T cell activation. Whereas SECTM1A acted as a co-stimulator of T cells, enhancing IL-2 production and proliferation, SECTM1B proved inhibitory to TCR mediated T cell activation. Surprisingly, both functional outcomes proved to be CD7-independent, indicating the existence of alternative receptors for both ligands. We used a SECTM1A-Fc fusion protein to immunoprecipitate potential alternative ligands from detergent lysates of CD7^−/−^ T cells and, using mass spectrometry, identified GITR as a SECTM1A binder. SECTM1A was found to bind to activated CD4+ and CD8+ T cells as well as to CHO cells expressing cell surface GITR. Binding of SECTM1A to activated primary T cells was inhibited by either GITRL-Fc or anti GITR antibodies. Thus SECTM1A and SECTM1B represent novel reciprocal alternative ligands which may function to modulate the activation of effector and regulatory T cells. The ability of SECTM1A to activate T cells may be explained by its ability to bind to GITR.

## Introduction

T cell responses to antigen are controlled at multiple levels. These include control of the cells' anatomical location, local MHC-antigen concentration on APCs, intracellular signalling changes, inhibition by regulatory T cells and soluble and cell surface regulatory ligand molecules expressed by antigen presenting cells. Examples of the latter group include receptor/ligand pairs such as GITR and GITR ligand, previously described by our laboratory to control activation of effector and regulatory T cells [1] PD1/PDL, CTLA4/B71&2, 41BB/41BBL, ICOS/ICOS ligand and CD7/SECTM1B (Secreted and transmembrane1B). CD7 is a T cell and NK cell expressed 40 kD transmembrane protein of the immunoglobulin superfamily which can induce lipid and tyrosine kinase activity and is thought to be important for T/NK cell activation [2].

A report of aberrant Treg development and function in CD7/CD28 double knockout mice [3], and our observation of elevated CD7 expression by TGFβ-induced regulatory T cells (unpublished observations) prompted us to investigate the effects of known CD7 ligands on T cell responses. Human and murine CD7 have previously been shown to interact with cell surface and secreted members of the immunoglobulin superfamily named SECTM1 (K12) and SECTM1B respectively [4].

Murine SECTM1B identified by Lyman et al [4] had been shown to inhibit proliferation of ConA-stimulated T lymphocytes whilst up regulating activation markers on activated NK cells. The human genome contains only one SECTM gene, SECTM1 which is situated in close proximity to CD7 on chromosome 17q25. As described here the mouse genome contains two SECTM genes in close proximity to the CD7 gene. Mouse SECTM1A shares the greatest homology to human SECTM1. SECTM1-Fc fusion proteins were shown by Lyman and colleagues to increase surface activation markers on human NK cells [4]. Wang et al reported SECTM1-Fc fusion proteins co-stimulated IL-2 and interferon gamma production by human CD4+ and CD8+ cells [5]. SECTM1 is predicted to be a type 1A transmembrane protein with extracellular N terminus and cleaved signal peptide. Depending on the experimental system used, SECTM1 appears either as a Golgi-associated protein which is secreted as a 20 kDa form, in breast carcinoma cells [6] or as a plasma membrane expressed surface protein in COS-1 cells transfected with expression plasmids [4]. SECTM1 is predominantly expressed by cells of the myeloid lineage and epithelia where its expression is enhanced by exposure to interferon gamma in a Stat-1 dependent manner [7]. It is possible that its ultimate location, secreted or on the plasma membrane, is dependent on either the cell on which it is expressed, the tissue location or micro environmental factors such as inflammation.

In this study we report the function of the novel murine CD7 ligand SECTM1A whose gene is located in the CD7/SECTM1B locus on mouse chromosome 11 with a tissue distribution differing from the known CD7 ligand SECTM1B. We constructed fusion proteins between the extracellular domains of SECTM1A and SECTM1B with an aglycosyl mutant Fc region of human IgG1, or anchored forms expressed on the cell surface of CHO cells. These were used to probe the function of these ligands in the experiments described here. Exposure of T cells to SECTM1A or SECTM1B resulted in reciprocal modulation of T cell proliferation. Surprisingly, despite SECTM1A being a ligand for CD7, all of these signalling events turned out to be CD7 independent. The co-stimulatory functions of SECTM1A may be explained in part by our observation that it is able to interact, albeit weakly with GITR as an alternative receptor.

## Results

### The identification of mSECTM1A as a novel mCD7 ligand with homology to hSECTM1 and mSECTM1B

**Figure 1:**
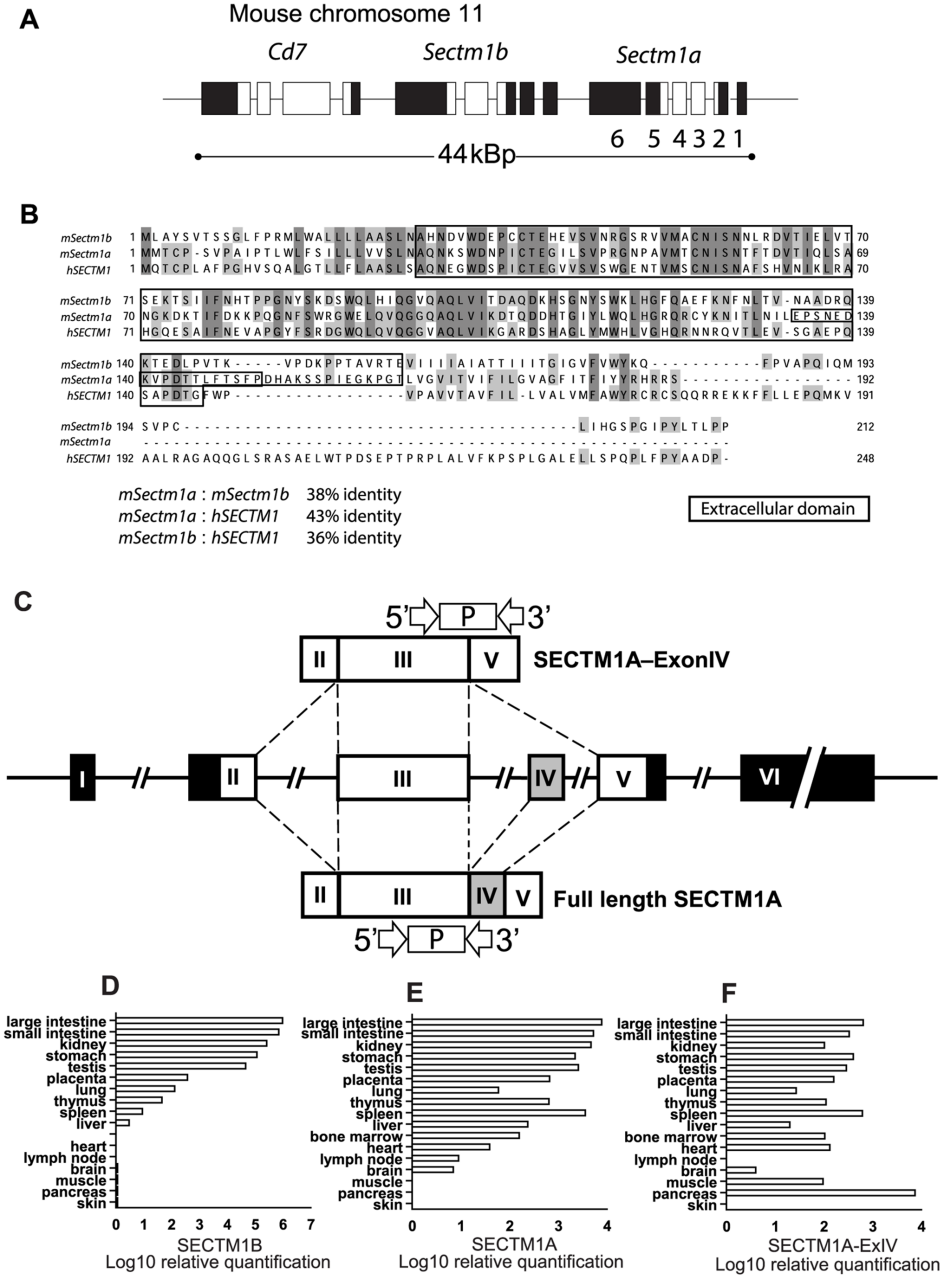
Location, identity and tissue distribution of SECTM1A. A**.** Chromosomal organisation of the *Cd7*, *Sectm1b* and *Sectm1a* genes. **B.** Alignment of *Sectm1a*, *Sectm1b* and human *SECTM1* was performed in the Jalview program using the MUSCLE algorithm [31]. Shading indicates the degree of amino acid identity between the human SECTM1 and mouse SECTM1A and SECTM1B. Dark grey indicates a residue conserved in all three sequences. Light grey indicates a residue shared by only two sequences. No shading indicates areas of divergent sequence. The boxed region within the extracellular region of SECTM1A indicates the peptide encoded by exon IV **C.** Positions of the taqman primers and probes used to quantify expression of the different splice variants of SECTM1A. **D.** Tissue distribution of *Sectm1b*, **E**. *Sectm1a* (full length) **F.** *Sectm1a* –ExIV transcripts. mRNA was quantified using the taqman primers and probes illustrated in Figure 1A. Samples were normalised to the level of *Sectm1b* in brain which was given an arbitrary value of 1.

The gene previously described as the murine orthologue of human *SECTM1*, murine *Sectm1b* [4], and *Sectm1a*, a newly annotated gene encoding an uncharacterised protein, lie in a 44kbp region neighbouring CD7 (Figure 1A). Alignment of the amino acid sequences of SECTM1A, SECTM1B and human SECTM1 reveals moderate homology between the three proteins in their extracellular regions, the greatest homology being between mouse SECTM1A and human SECTM1 which share 43% amino acid identity (Figure 1B). Cloning of *Sectm1a* from splenic cDNA revealed a major splice variant lacking exon IV (data not shown). Exon IV encodes a stalk region joining the immunoglobulin domain to the transmembrane domain (Figure 1C). Tissue distribution of *Sectm1a*, *1a-ExIV* and *Sectm1b* transcripts was assessed by real-time RT-PCR (Figure 1D-F). Primers and probes used for this analysis are shown diagrammatically in figure 1C. *Sectm1b* had a variable distribution with the highest expression detectable in large and small intestine and being undetectable in bone marrow and lymph nodes (Figure 1D). Primers spanning exons 3 and 4 were used to measure full length *Sectm1a* only (Figure 1E) whereas those spanning exons 3–5 detect *1a-ExIV* (Figure 1F). *Sectm1a* and *1a-Exon IV* were more widely expressed than *sectm1b*.

**Figure 2:**
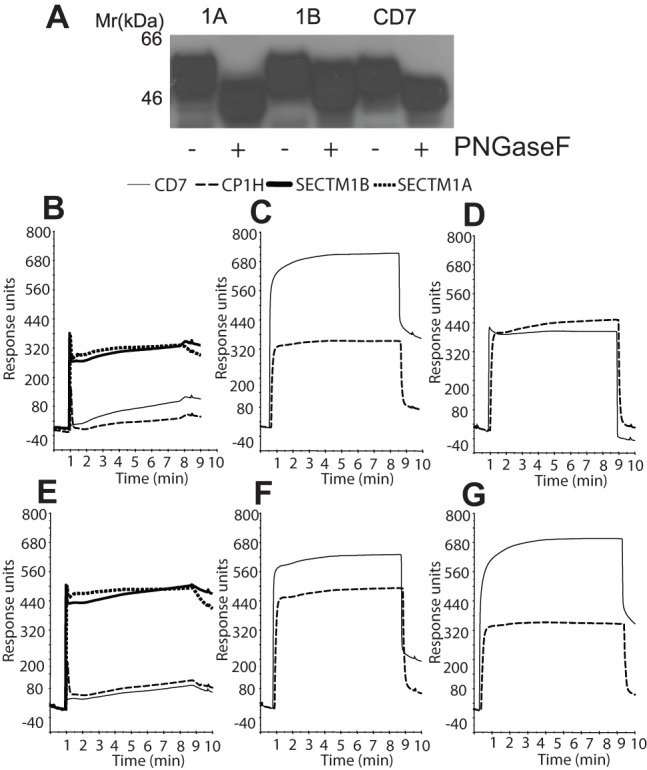
SECTM1A binds to CD7. A**.** Western blot of mSECTM1A, SECTM1B and CD7 Fc fusion proteins digested with protein N glycosidase F (PNGaseF). Biotinylated anti-human Fc antibody was used to western blot the resultant digested proteins. Representative blot of two shown. **B.** Analysis of mSECTM1A and mSECTM1B Fc fusion proteins binding to mCD7-Fc. CD7-Fc was passed over flow cells coated with CD7-Fc, SECTM1B-Fc, SECTM1A-Fc and CAMPATH-1H in a BIAcoreTM. Results in B-G representative of three separate experiments. **C**. SECTM1B-Fc binds solid phase CD7-Fc. SECTM1B-Fc was passed over CD7-Fc. **D**. SECTM1A-Fc was passed over the same flow cell as in (C) directly after SECTM1B-Fc to detect binding to CD7-Fc after binding to SECTM1B-Fc. **E.** On a separate CM5 chip CD7-Fc was passed over flow cells coated with CD7-Fc, SECTM1B-Fc, SECTM1A-Fc and CAMPATH-1H as in 2B **F.** SECTM1A-Fc binds solid phase CD7-Fc. SECTM1A-Fc was passed over CD7-Fc. **G.** SECTM1B-Fc was passed over the same flow cell as in (F) directly after SECTM1A-Fc to detect binding to CD7-Fc after binding to SECTM1A-Fc.

To probe the functions of murine SECTM1A/B we constructed fusion proteins between the extracellular domains of SECTM1A, SECTM1A-ExIV, SECTM1B and CD7 with the aglycosyl mutated Fc portion of human IgG1. Full length SECTM1A-Fc fusion proteins were relatively labile therefore SECTM1A-ExIV was used in most subsequent investigations (termed “SECTM1A” from now on). SECTM1A, SECTM1B and CD7, are predicted to have 4, 3 and 3 N-linked glycosylation sites respectively. Digestion with peptide N-glycosidase F revealed a shift in migration by SDS PAGE confirming that the Fc-fusion proteins were indeed glycosylated (Figure 2A).

We used surface plasmon resonance to test the functionality of the SECTM fusion proteins and to test the hypothesis that SECTM1A might be an alternative ligand for CD7. CD7, SECTM1A and SECTM1B-Fc-fusion proteins were immobilised onto BIAcoreTM CM5 chips and the same proteins were injected over the flow cells in solution to test binding. CD7-Fc in solution caused a rapid increase in response units indicating fast binding to both SECTM1A and SECTM1B in a reproducible manner (bold solid and dotted lines, Figure 2B &E). Thus SECTM1A represents a novel alternative ligand for mouse CD7.

CD7-Fc dissociated from SECTM1A more rapidly than SECTM1B suggesting that CD7 binds with higher affinity to SECTM1B than SECTM1A (Figure 2B and 2E, dissociation at 8–9 minutes bold solid and dotted lines). We then tested whether SECTM1B could block binding of SECTM1A to CD7. SECTM1B-Fc was passed over solid phase CD7 and bound rapidly (Figure 2C, solid line). When SECTM1A was passed over the same flow cell directly after SECTM1B no binding above background was observed (Figure 2D, solid line). Thus SECTM1B competes for CD7 binding with SECTM1A. In a reciprocal experiment, SECTM1A was passed in solution over solid phase CD7 (Figure 2F, solid line) and binding was observed albeit with less increase in response units seen for SECTM1B. Directly following SECTM1A, SECTM1B was passed over the same flow cell. This time binding to CD7-Fc was observed with almost exactly the same stoichiometry as when SECTM1B bound CD7-Fc alone (Figure 2G, solid line) which is consistent with a weaker affinity of SECTM1A for CD7. SECTM1A and SECTM1B did not exhibit homophilic binding nor did they bind to each other under these conditions.

### SECTM1B inhibits activation and IL-2 production of T cells

**Figure 3:**
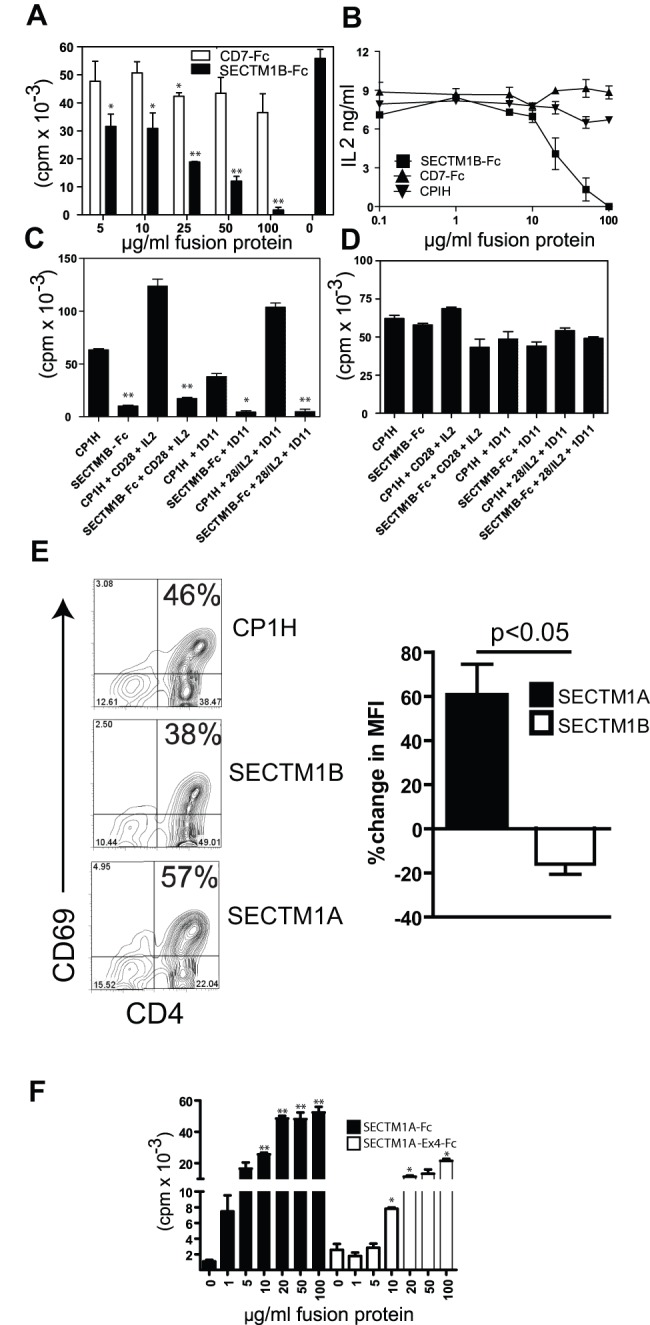
SECTM1A costimulates whereas SECTM1B inhibits T cell activation. A. 48+ T cells cultured on 2C11 coated plates in the presence of solid-phase mCD7-Fc or mSECTM1B-Fc fusion protein. Campath-1H antibody was used to equilibrate each well to an equal protein concentration. Results representative of five separate experiments. * = p<0.05, ** = p<0.005 by Student's t test. **B.** ELISA for IL-2 in supernatants of CBA/Ca CD4+ T cells stimulated for 48 hours in the presence of plate-bound 2C11 and solid phase SECTM1B-Fc, CD7-Fc or Campath-1H. Results are representative of three experiments. SECTM1B inhibition significant at 20,50 and 100 ug plated fusion protein assessed by Student's t test. **C.** Proliferation assay with CD4+ T cells cultured on 2C11 coated plates in the presence of solid-phase mSECTM1B-Fc fusion protein or Campath-1H as control. Anti-mCD28 or neutralising anti-TGFβ (1D11) was added to some wells at 1 ug/ml. Where indicated, IL-2 was added at 10 U/mL. * = p<0.05, ** = p<0.005 by Student's t test. Results are representative of two experiments. **D.** Proliferation assay with CD4+ T cells. The supernatants from the cell cultures described in ‘Figure C’ were added to fresh cultures of CBA.CA CD4+ T cells cultured with plate-bound 2C11. Proliferation was assessed at 48 hours. Results representative of two separate experiments. Differences not significant by Student's t test. **E**. Left panel; Cell surface expression of CD69 on CD4+ T cells (approximately 85% pure) cultured for 48 hours on 2C11(1 ug/ml) coated 96 well plates in the presence of plate-bound CP1H, SECTM1B-Fc or SECTM1A-Fc. All fusion proteins coated at 10 ug/ml. Results representative of three separate experiments. Right Panel; Pooled data from three experiments showing percentage change in CD69 on activated CD4+ T cells. Cells were cultured as above in the presence of 10 ug/ml plate bound fusion proteins and the percentage change in mean fluorescence intensity for CD69 above or below the CP1H control is shown. Statistical significance was measured using Student's T test. **F.** Proliferation assay with CD4+ T cells cultured on 2C11 coated plates in the presence of solid-phase mSECTM1A-Fc fusion protein or SECTM1A-ExIV-Fc. Proliferation was measured at 48 hours. Results representative of five separate experiments. * = p<0.05, ** = p<0.005 by Student's t test.

It is unclear what functions SECTM1A and SECTM1B serve. Our preliminary experiments with SECTM1B-Fc showed no evidence for modulation of T cell responses when the fusion protein was used in solution (data not shown). However solid phase SECTM1B-Fc but not CD7-Fc inhibited both IL-2 production and proliferation of T cells activated through anti-CD3 stimulation (Figure 3 A & B). The inhibitory effect was not due to steric hindrance of the anti-CD3 antibodies as equal protein was loaded onto each culture well using Campath-1H (anti human CD52 with the same human IgG1, aglycosyl Fc fragment as the fusion proteins) as an irrelevant aglycosyl human IgG1 antibody ‘filler’. The effects of SECTM1B were not due to induction of apoptosis as shown by trypan blue exclusion and annexin V staining (data not shown). Inhibition of T cell activation by SECTM1B occurred in the presence of co-stimulation and exogenous IL-2 (Figure 3C) and was independent of TGFβ as addition of neutralising anti-TGFβ (1D11, [8]) did not reverse this effect. We then tested whether the mechanism of SECTM1B inhibition relied on soluble inhibitory factors being released into the cell culture supernatant. Culture supernatants from the experiment shown in Figure 3C were transferred to fresh cultures of anti-CD3 stimulated CD4+ T cells (Figure 3D) showing equivalent proliferation of T cells grown for 48 hours in the presence of each supernatant. Thus this does not appear to be a major mechanism, however we cannot exclude the possibility that soluble inhibitory factors may be produced which immediately bind to the T cells, effectively sequestering them out of the culture supernatants.

### SECTM1A is co-stimulatory for T cell activation

We compared the effects of SECTM1A and SECTM1B on T cell activation. First we compared the level of the activation marker CD69 expressed on the surface of CD4+ T cells following 48 hours of stimulation with immobilised anti-CD3 in the presence of immobilised SECTM1A or SECTM1B. Following activation in this manner SECTM1B reduced the percentage of cells expressing cell-surface CD69 (Figure 3E). Surprisingly CD4+ T cells activated in the presence of SECTM1A showed an increase of CD69 over those cultured in the presence of the control protein. Thus the cell surface phenotype of cells stimulated in the presence of SECTM1B or SECTM1A was consistent with inhibitory or co-stimulatory signalling respectively.

Both splice variants of SECTM1A, full length and lacking exon IV, enhanced T cell activation. CD4+ T cells activated in the presence of the full length ligand or the truncated isoform lacking exon IV also showed enhanced proliferation with increasing concentrations of plate-bound SECTM1A (Figure 3F).

**Figure 4:**
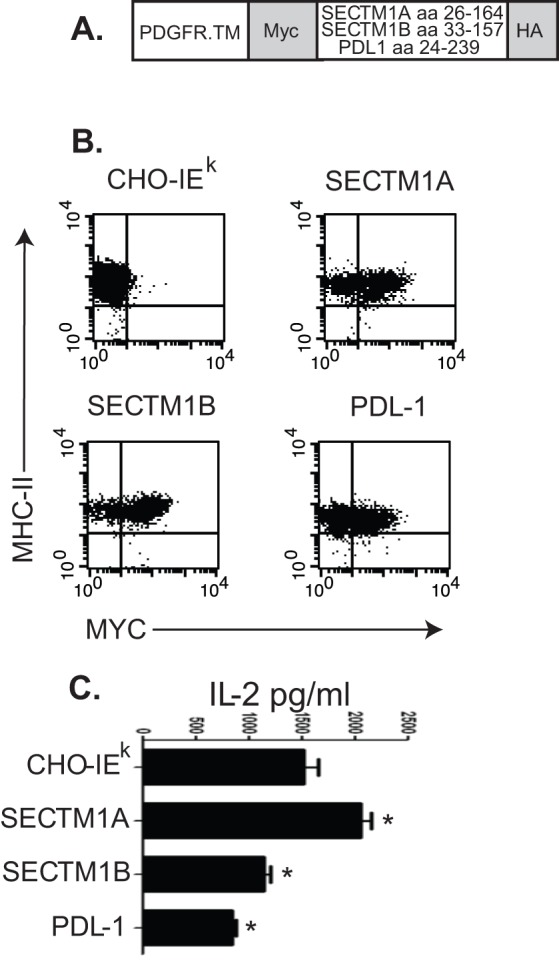
Cell surface targetted SECTM1A enhances IL-2 production by antigen specific T cells. A. Diagram of the structure of pDISPLAY based constructs for cell surface expression of SECTM1A/B and PDL1 on CHO cells expressing MHC class II ‘CHO-IE^k^’. The regions of SECTM1A/B and PDL1 between the leader sequence and plasma membrane transmembrane sequence were used. **B.** Flow cytometry for MHC class II and Myc (to detect cell surface SECTM1A/B or PDL1) on CHO-IE^k^ cells. **C.** ELISA measurement of IL-2 in 48 hour co-culture of male peptide-pulsed CHO-IE^k^ cells and A1.RAG T cells. Representative of two separate experiments. * = p<0.05 by Student's t test.

We then investigated whether SECTM1A and B are co-stimulatory or co-inhibitory respectively when situated on the plasma membrane of an antigen presenting cell. In order to reduce the contribution from other co-stimulation molecules we used CHO cells which were stably transfected with MHC class II (IE^k^). We transfected these cells with membrane targeted constructs in the vector pDISPLAY (Figure 4A) which targets Myc and HA tagged fusion proteins, the extracellular domains of SECTM1A, B or PDL-1 in this case, to the plasma membrane via the transmembrane domain of the platelet derived growth factor receptor (PDGFR, Figure 4B). CD4+ T cells from female A1.RAG^−/−^ TCR transgenic mice, whose T cells recognise the male antigen, were cultured in the presence of CHO.IE^k^ cells expressing the SECTM1 constructs pulsed with male antigen. Following 48 hours of culture A1.RAG^−/−^ T cells stimulated in the presence of CHO cells expressing SECTM1A secreted significantly more IL-2 and those cultured in the presence of CHO cells expressing SECTM1B significantly less IL-2 (Figure 4C). Thus SECTM1A and B represent co-stimulatory and inhibitory receptors in the context of MHCII/peptide mediated T cell activation.

**Figure 5:**
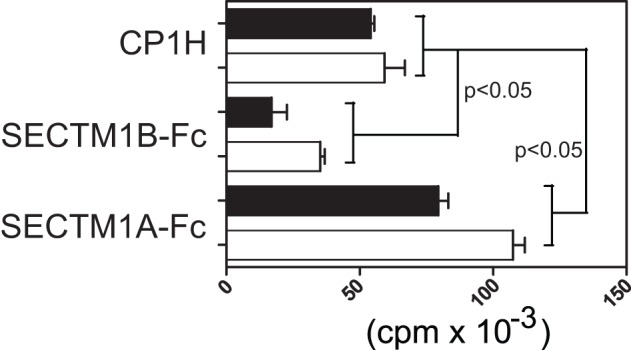
Modulation of T cell proliferation by SECTM1A and SECTM1B occurs independently of CD7. Proliferation assay with C57BL/6 (black bars) and CD7^−/−^ (white bars) CD4+ T cells cultured on 2C11 coated plates in the presence of 10 μg solid-phase mSECTM1A-Fc fusion protein or SECTM1B-Fc. Proliferation was measured at 48 hours. Results representative of five separate experiments. Statistical significance determined by Student's t test.

One explanation for opposing functional outcomes from SECTM1A and B is that although both bind CD7, as detected by BIAcore, they may have additional alternative receptors on T cells. To test this hypothesis we used CD4+ T cells from C57BL/6 CD7^−/−^ mice and found (Figure 5) that remarkably, in in vitro stimulation assays, both SECTM1A-mediated co-stimulation and SECTM1B mediated inhibition of T cell proliferation occur in the absence of CD7. This indicates that there must indeed be additional receptors, other than CD7, for both these ligands on T cells.

**Figure 6:**
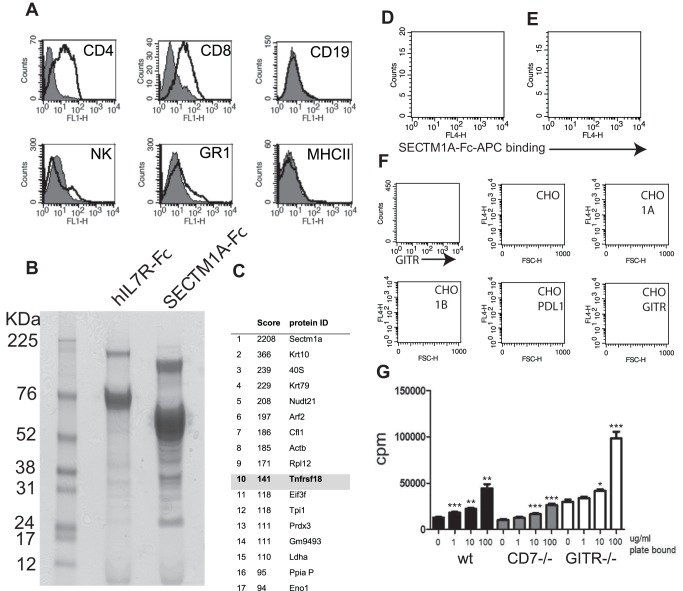
SECTM1A binds to GITR. **A.** SECTM1A binds to the surface of activated CD4+ and CD8+ T cells. Flow cytometry of SECTM1A-Fc binding to 48 hour concanavalin A-activated CD7^−/−^ splenocytes. Cells were double stained for the indicated markers (CD4, CD8, CD19, NK1.1, GR1 and MHCII) and APC-conjugated SECTM1A-Fc. Filled histograms represent background staining with a control human IgG1 aglycosyl Fc region conjugated to APC. Clear histograms represent staining with SECTM1A-Fc conjugated to APC. Results representative of two experiments. **B.** Coomassie blue stained SDS-PAGE gel showing immunoprecipitation eluates from Lauryl maltoside lysates of concanavalin A-activated CD7^−/−^ splenocytes bound to hIL7R-Fc (negative control) and SECTM1A-Fc/protein-G agarose columns. Major bands at 76kDa (IL7R-Fc) and 55 kDa (SECTM1A-Fc) are Fc fusion proteins eluted off the immunoprecipiatation column. **C.** Identities of proteins eluted from the gel in (B) with a MASCOT score of >90. **D.** APC conjugated SECTM1A-Fc binding to anti-CD3 and anti-CD28 activated CD7^−/−^ CD4+ T cells. Clear histogram, negative control; binding to cells pre-incubated for 1 hour with Rat IgG2b (isotype control). Shaded histogram, binding to cells preincubated with rat anti-GITR (YGITR-765). Results representative of two separate experiments. **E.** APC conjugated SECTM1A-Fc binding to anti-CD3 and anti-CD28 activated CD7^−/−^ CD4+ T cells. Clear histogram, binding to cells pre-incubated for 1 hour with hIL7R-Fc (negative control). Shaded histogram, binding to cells preincubated with GITRL-Fc. Results representative of two separate experiments. **F.** SECTM1A binds to CHO cells expressing cell surface GITR. Binding of APC conjugated SECTM1A-Fc to CHO cells expressing plasma membrane-targetted SECTM1A, SECTM1B, PDL1 or GITR driven by pDISPLAY constructs. Top left panel indicates the level of cell surface GITR expression by the CHO-GITR cells, staining with YGITR765 (thin line), thick line represents isotype staining control. Dot plots represent SECTM1A-Fc binding. Text on dot plots indicates the cell type being stained. **G.** SECTM1A costimulates CD4+ T cells lacking GITR expression. CD4+ T cells from C57BL/6, CD7^−/−^ and GITR^−/−^ mice were activated for 48 hours in the presence of plate-bound anti-CD3 (145-2C11) and the indicated amounts of plate-bound SECTM1A-Fc. Cell division was measured using tritiated thymidine incorporation. * = p<0.05, ** = p<0.005, *** = p<0.0005by Student's t test.

To identify potential alternative SECTM1 ligands we first determined the cell types expressing the ligands. To this end we used allophycocyanin-labelled SECTM1A and SECTM1B Fc fusion proteins in FACS to stain CD7^−/−^ resting and conA activated splenocytes. Despite its potent inhibitory effects on T cell activation in the solid phase, SECTM1B-Fc did not bind detectably to wt or CD7^−/−^ resting or activated splenocytes in this FACS assay (data not shown). However SECTM1A-Fc was shown to bind to activated CD4+, CD8+ and to a lesser extent NK cells and granulocytes from CD7^−/−^ mice (**Figure** **6A**).

As we were able to demonstrate binding of SECTM1A to activated CD7^−/−^ CD4+ T cells by FACS, we used lauryl maltoside lysates of concanavalin A-activated CD7^−/−^ splenocytes to immunoprecipitate the potential ligand(s) as described in *methods.* Eluates from SECTM1A-Fc/protein G agarose columns contained multiple proteins of different molecular weights (figure 6B). Coomassie stained gel slices containing the eluted proteins were tryptically digested and subjected to MS/MS mass spectroscopy for identification. Peptides with a MASCOT score greater than 90 are shown in figure 6C. The only protein with a plasma membrane transmembrane segment other than SECTM1A-Fc itself which was derived from the immunoprecipitation procedure was TNFRSF18 (GITR).

To confirm the SECTM1A/GITR interaction we tested whether binding of SECTM1A to activated CD7^−/−^ CD4+ T cells could be inhibited by pre-incubation of the cells with either anti-GITR antibodies or GITRL. As shown in figure 6D and 6E both anti-GITR and soluble GITRL led to a reduced intensity staining of cells by SECTM1A-Fc. The specificity of the interaction was confirmed by utilising CHO cells expressing SECTM1A, SECTM1B, PDL-1 or GITR as targets for FACS staining with SECTM1A-Fc (Figure 6F). Only CHO cells expressing GITR gave a substantial signal in this FACS assay. Finally, we tested whether SECTM1A costimulates T cells predominantly via GITR, or CD7. We activated splenic CD4+ T cells isolated from wild type, CD7^−/−^ and GITR^−/−^ mice with plate bound anti-CD3 in the presence of increasing concentrations of plate-bound SECTM1A-Fc. We showed that SECTM1A-Fc increases the proliferation induced by anti-CD3 of T cells from all three mice. Thus SECTM1A can costimulate via CD7, GITR and/or possible other unidentified ligands.

## Discussion

In this study we have demonstrated a novel mode of immune regulation via the opposing actions of two murine immunoglobulin superfamily proteins SECTM1A and SECTM1B. These widely expressed plasma membrane proteins modulate activation of CD4+ T cells. We have demonstrated binding of both proteins to CD7 with surface plasmon resonance; however, they can exhibit *in vitro* functions independently of CD7. In the case of SECTM1A this may be explained by its ability to bind to GITR in the absence of CD7.

We observed significant differences in transcript expression between tissues. SECTM1B had a more restricted distribution than SECTM1A. SECTM1B transcripts were undetectable in bone marrow, heart, lymph nodes, brain, muscle, pancreas and skin whereas SECTM1A transcripts were detectable in these tissues although there were differences between SECTM1A and the spliced form lacking exon IV. It may be that that the presence of only SECTM1A in lymph nodes might help to promote T cell priming whereas the balance of SECTM1A/B in peripheral tissues could promote or inhibit T cell responses. Analysis of potential peptidase sites in the exon IV region of the SECTM1A protein reveals four potential cleavage consensus sites for glutamyl and aspartic endopeptidases which may generate soluble forms of the protein from the full length precursor (data not shown). It remains to be shown whether the spliced transcript of SECTM1A lacking exon IV could serve to provide a stable membrane-anchored form of the protein whilst the full length form could act as a precursor of a soluble form of the protein.

Our Biacore data clearly show that SECTM1B has a higher affinity for CD7 than SECTM1A and this hierarchy of binding may also extend to the alternative SECTM receptors on T cells. Differences in functional effects suggest that the differences in affinity for CD7 may apply to binding to these alternative receptors. Although we were unable to identify the alternative SECTM1B receptor in this study it is possible that both SECTM1A and B share other unidentified alternative receptors. An alternative explanation for the reciprocal effects of SECTM1A and B may be due to differences in efficacy of blocking rather than crosslinking of the same receptor. Monovalent binding by the higher affinity reagent SECTM1B-Fc would result in blocking whereas the lower affinity SECTM1A-Fc would be an effective crosslinking reagent resulting in activation. Similar effects of affinity and valency have been noted before and quantitatively analyzed [9].

SECTM1A is an immunoglobulin-like receptor whereas GITR is a member of the TNFR superfamily and is known to bind GITRL, a member of the TNF superfamily ligand group. All other TNFR superfamily members bind to ligands of the TNF superfamily. The only known exception to this rule is that of Herpes virus entry mediator (HVEM, TNFRSF14) which was shown to bind to B and T Lymphocyte Attenuator (BTLA, CD272) [10], [11] an immunoglobulin superfamily member, on T cells, in addition to four other ligands, LIGHT, CD160, lymphotoxinα and Herpes simplex virus glycoprotein D [12]. The binding of HVEM to BTLA on T cells is inhibitory to T cell activation [10].

TNF family ligands form non-covalent homotrimers which induce the receptor to adopt a trimeric conformation which enhances signalling [13]. In the case of the BTLA-HVEM interaction it has been shown that a 1∶1 stoichiometry can exist between extracellular domains of BTLA and HVEM however BTLA exists as oligomers on the cell surface which may be capable of clustering HVEM to enhance signalling [14], [15]. Human GITRL has also been shown to adopt a homotrimeric conformation [16] inducing homotrimeric clusters of GITR on the cell surface. Mouse and human GITRL differ in their propensity to form trimers in solution with mGITRL forming stable dimers [17]. The stoichiometry of the interaction between GITR and SECTM1A seen in our studies is currently unknown. Despite our demonstration that both SECTM1A-Fc and GITR-Fc fusion proteins are active in biochemical and cell binding experiments we did not detect a specific interaction between them by surface plasmon resonance.

Whether binding to CD7 or to GITR it is possible that ‘reverse’ signalling occurs within the cells expressing cell surface SECTM1A or SECTM1B, which include epithelial, endothelial and myeloid cells. Both proteins have short cytoplasmic tails containing tyrosine residues which do not conform to known signaling motifs. The possibility of reverse signalling remains to be excluded.

Functionally it has been reported that murine SECTM1B inhibits ConA-induced lymph node cell proliferation but not lymph node cell proliferation induced by anti-αβ TCR antibodies [4]. We consistently observe inhibition of anti-CD3 induced T cell proliferation and IL-2 production with SECTM1B, and the converse with SECTM1A. These differences may be due to differences in cell preparation as we used highly purified negatively selected CD4+ cells compared to the previously published study.

The SECTM proteins join a large group of ligands used by tissues to co-stimulate or diminish T cell responses [18], [19]. [1], [20], [21], [22], [23], [24], [25], [26] [27]. Co-expression of the SECTM molecules in tissues may have alternate outcomes depending on the receptors expressed by different T cells. A degree of tissue regulation of SECTM1 has been shown by Lam and colleagues who demonstrated up-regulation of SECTM1 expression in response to IFNγ by thymic epithelium [28].

In conclusion we have demonstrated opposite functional outcomes of SECTM1A and SECTM1B, widely expressed tissue ligands, binding to T cells. We have shown that SECTM1A represents a new CD7 ligand in mice in addition to binding to GITR and we raise the possibility that alternative receptors for both SECTM1A and SECTM1B exist on T cells.

## Materials and Methods

### Mice

A1(M).RAG−/−, CBA/Ca, C57BL/6 and C57BL/6.CD7−/− mice were bred and maintained in SPF conditions at the Sir William Dunn School of Pathology. CD7−/− mice were the kind gift of Dr. Barton Haynes, Duke University. All procedures were conducted in accordance with the Home Office Animals (Scientific Procedures) Act of 1986 and received approval from the local ethical review panel at the University of Oxford.

### Cells and antibodies

‘Untouched’ splenic CD4+ T cells were isolated by negative selection via magnetic sorting using commercial kits (Miltenyi Biotech). Anti-CD4, CD25, CD8, CD19, NK1.1, GR1, MHCII, CD69 and CD62L were obtained from BD Biosciences. FITC conjugated anti-Myc (9E10) was purchased from Sanatacruz Biotechnology. Anti-CD3 (145.2C11), anti-CD28 (37.51) anti-GITR (YGITR765.4.2), anti MHCII (YTA94.8.10) and neutralizing anti-TGFβ (1D11) were produced from hybridoma supernatants in house.

CHO cells stably transfected with MHC class II (IE^k^) were a kind gift from Prof Neil Barclay, Sir William Dunn School of Pathology Oxford.

### Quantitative real-time RT-PCR

Taqman RT-PCR was performed essentially as described [29]. Primers and probes were purchased from Eurogentech: SECTM1B, 5′-AAGCAGCTGGGTTCTTCGGT-3′, 5′-TGGACACCA TGATCAGATGACAA-3 and FAM-5′-TGAACTGAATGGGAAGAAGAGAACA GAGCATTC-3′-TAMRA. Primers spanning the junction of SECTM1A exons 3–4 were assayed using a commercial primer/probe set(Applied Biosystems Mm00520313_g1). SECTM1A exons 3–5 junction was assayed using 5′-GACGACCACACAGGGATATACTTG-3′, 5′-CTCTTCGCGTGATCTAAGATATTC AG-3′ AND FAM-5′-CATGGACGCCAGAGATGCTACAAAAACA-3′-TAMRA.

### Production of SECTM1 Fusion proteins

Fc fusion protein expression plasmids were constructed using an expression vector pEE12(CMV/T7)CD5Lhγ1 encoding a CMV promoter and CD5 leader sequence directly 5′ to the human IgG1 aglycosyl Fc coding region. cDNAs were cloned between the CD5 leader sequence and hIgG1 fragment.

Fusion proteins were produced in CHO-S cells and purified with protein G agarose with elution using glycine at pH 2.0.

### Protein N glycosidase F digestions

20 μg of Fc fusion proteins were denatured and subjected to digestion with 1000 units of protein N glycosidase F (New England Biolabs) for 2 hours at 37°C according to the manufacturers instructions. Digested and un-digested fusion proteins were run on SDS PAGE, coomassie stained and western blotted for human Ig-Fc to assess their level of N-linked glycosylation.

### Proliferation assays

CD4+ T cells were stimulated with plate-bound anti-CD3 (145.2C11) and IL-2 or anti CD28 where indicated. SECTM1A, 1A-ExIV, SECTM1B-Fc fusion proteins or Campath-1H as an Fc control with identical human IgG1 aglycosyl Fc region were immobilised by air-drying onto 96 well culture plates as described by Stebbings et al [30]. 1×105 CBA/Ca CD4+ T cells were used per well. Tritiated thymidine at 0.5 µCi per well was added sixteen hours before harvesting the cultures onto glass fibre filters and scintillation counting using standard techniques.

### ELISA

IL-2 was measured by ELISA using a paired antibody set from BD Biosciences and recombinant IL-2 from R&D Systems to construct standard curves.

### Flow cytometry

Fc fusion proteins used for FACS were labelled with allophycocyanin using a Lynx rapid conjugation kit (AbdSerotec) according to the manufacturer's instructions. Cells were incubated with fusion proteins at 10 ug/ml in PBS with 0.5% bovine serum albumin and 2 mM EDTA for 1 hour at 4C followed by three PBS washes and fixation in 2% paraformaldehyde.

### Surface Plasmon Resonance Analysis

For surface plasmon resonance experiments a BIAcore 3000 instrument was used at 25°C. Rabbit anti-mouse antibody BR-1000-57 (BIAcore) was directly immobilized by amine coupling in 10 mM NaAc buffer, pH 5.5 onto CM5 chips. This was followed by binding of mouse anti human IgG (SBH2, a kind gift from Alison Tutt, Tenovus, Southampton) to all cells. CAMPATH-1H, mCD7-Fc, hIL7R-Fc, SECTM1A-Fc, SECTM1B-Fc, GITR-Fc and GITRL-Fc were bound to separate flow cells. Fc-fusion protein-containing solutions at 25 µM were injected in HEPES-buffered saline, pH 7.4, over the immobilized capture antibody-Fc fusion protein containing flow cells at a rate of 5 µl per minute.

### Immunoprecipitation of SECTM1A interactors

5 mg of SECTM1A-Fc or hIL7R-Fc negative control fusion protein were incubated in PBS with 1 ml of protein G agarose (Invitrogen) for 1 hour at 4C. The agarose was then washed with PBS followed by 50 mM sodium borate and the fusion proteins cross-linked to the protein G with dimethyl pimelimidate at 7 mg/ml in 0.2 M triethanolamine for 1 hour at room temperature. The columns were washed with 5 column volumes of 0.2 M triethanolamine followed by blocking with 5 column volumes of 0.1 M ethanolamine. Un-cross-linked fusion proteins were eluted from the column with 0.2 M glycine pH2.0. 2×10^8^ concanavilin-A-activated CD7^−/−^ splenocytes were lysed on ice with 0.5% lauryl maltoside prior to clearing by centrifugation. Lysates were incubated with the columns for 2 hours at 4C followed by washing with 5 column volumes of PBS and elution with 0.2 M glycine. Eluates were concentrated with Centricon 10,000 molecular weight cutoff dialysis spin columns (Millipore) and run on SDS PAGE gels.

### Mass Spectroscopy

SDS-PAGE gel slices were digested with trypsin and desalted on a C18 packed pipette tip. Samples were injected onto an Ultimate 3000 nano HPLC (Dionex) system coupled to an Orbitrap mass spectrometer (Thermo Electron). Samples were resolved on a 5 cm by 100 micron inner diameter picotip column (New Objective) which was packed in house with reprosil-Pur C18-AQ phase. A 40 minute gradient was used to separate the peptides. The mass spectrometer was operated in data dependent acquisition mode. Precursor scans were performed in the orbitrap at a resolving power of 60,000, from which five precursor ions were selected and fragmented in the linear ion trap. Charge state +1 ions were rejected. Peak lists were generated using DTAsupercharge and searched using Mascot (Matrixscience). Data were searched against MSDB database, restricting the taxonomy to *mus musculus.* Precursor mass accuracy tolerance was set at 10 ppm and MS/MS at 0.5 Da.

## References

[1] Tone M, Tone Y, Adams E, Yates SF, Frewin MR, et al (2003) Mouse glucocorticoid-induced tumor necrosis factor receptor ligand is costimulatory for T cells. Proc Natl Acad Sci U S A 100: 15059–15064. 14608036 10.1073/pnas.2334901100 PMC299905

[2] Rabinowich H, Pricop L, Herberman RB, Whiteside TL (1994) Expression and function of CD7 molecule on human natural killer cells. J Immunol 152: 517–526. 7506726

[3] Sempowski GD, Cross SJ, Heinly CS, Scearce RM, Haynes BF (2004) CD7 and CD28 are required for murine CD4+ CD25+ regulatory T cell homeostasis and prevention of thyroiditis. J Immunol 172: 787–794. 14707048 10.4049/jimmunol.172.2.787

[4] Lyman SD, Escobar S, Rousseau AM, Armstrong A, Fanslow WC (2000) Identification of CD7 as a cognate of the human K12 (SECTM1) protein. J Biol Chem 275: 3431–3437. 10652336 10.1074/jbc.275.5.3431

[5] Wang T, Huang C, Lopez-Coral A, Slentz-Kesler KA, Xiao M, et al (2012) K12/SECTM1, an interferon-gamma regulated molecule, synergizes with CD28 to costimulate human T cell proliferation. Journal of leukocyte biology 91: 449–459. 22184754 10.1189/jlb.1011498 PMC3289399

[6] Slentz-Kesler KA, Hale LP, Kaufman RE (1998) Identification and characterization of K12 (SECTM1), a novel human gene that encodes a Golgi-associated protein with transmembrane and secreted isoforms. Genomics 47: 327–340. 9480746 10.1006/geno.1997.5151

[7] Huyton T, Gottmann W, Bade-Doding C, Paine A, Blasczyk R (2011) The T/NK cell co-stimulatory molecule SECTM1 is an IFN “early response gene” that is negatively regulated by LPS in human monocytic cells. Biochimica et biophysica acta 1810: 1294–1301. 21749909 10.1016/j.bbagen.2011.06.020

[8] Daley SR, Ma J, Adams E, Cobbold SP, Waldmann H (2007) A key role for TGF-beta signaling to T cells in the long-term acceptance of allografts. Journal of immunology 179: 3648–3654. 10.4049/jimmunol.179.6.3648 17785800

[9] Koch C, Staffler G, Huttinger R, Hilgert I, Prager E, et al (1999) T cell activation-associated epitopes of CD147 in regulation of the T cell response, and their definition by antibody affinity and antigen density. Int Immunol 11: 777–786. 10330283 10.1093/intimm/11.5.777

[10] Sedy JR, Gavrieli M, Potter KG, Hurchla MA, Lindsley RC, et al (2005) B and T lymphocyte attenuator regulates T cell activation through interaction with herpesvirus entry mediator. Nature immunology 6: 90–98. 15568026 10.1038/ni1144

[11] Murphy KM, Nelson CA, Sedy JR (2006) Balancing co-stimulation and inhibition with BTLA and HVEM. Nature reviews Immunology 6: 671–681. 10.1038/nri1917 16932752

[12] Sarrias MR, Whitbeck JC, Rooney I, Ware CF, Eisenberg RJ, et al (2000) The three HveA receptor ligands, gD, LT-alpha and LIGHT bind to distinct sites on HveA. Molecular immunology 37: 665–673. 11164894 10.1016/s0161-5890(00)00089-4

[13] Locksley RM, Killeen N, Lenardo MJ (2001) The TNF and TNF receptor superfamilies: integrating mammalian biology. Cell 104: 487–501. 11239407 10.1016/s0092-8674(01)00237-9

[14] Compaan DM, Gonzalez LC, Tom I, Loyet KM, Eaton D, et al (2005) Attenuating lymphocyte activity: the crystal structure of the BTLA-HVEM complex. The Journal of biological chemistry 280: 39553–39561. 16169851 10.1074/jbc.M507629200

[15] Nelson CA, Fremont MD, Sedy JR, Norris PS, Ware CF, et al (2008) Structural determinants of herpesvirus entry mediator recognition by murine B and T lymphocyte attenuator. Journal of immunology 180: 940–947. 10.4049/jimmunol.180.2.940 18178834

[16] Chattopadhyay K, Ramagopal UA, Mukhopadhaya A, Malashkevich VN, Dilorenzo TP, et al (2007) Assembly and structural properties of glucocorticoid-induced TNF receptor ligand: Implications for function. Proceedings of the National Academy of Sciences of the United States of America 104: 19452–19457. 18040044 10.1073/pnas.0709264104 PMC2148310

[17] Chattopadhyay K, Ramagopal UA, Brenowitz M, Nathenson SG, Almo SC (2008) Evolution of GITRL immune function: murine GITRL exhibits unique structural and biochemical properties within the TNF superfamily. Proceedings of the National Academy of Sciences of the United States of America 105: 635–640. 18182486 10.1073/pnas.0710529105 PMC2206588

[18] McAdam AJ, Schweitzer AN, Sharpe AH (1998) The role of B7 co-stimulation in activation and differentiation of CD4+ and CD8+ T cells [In Process Citation]. Immunol Rev 165: 231–247. 9850864 10.1111/j.1600-065x.1998.tb01242.x

[19] Sharpe AH, Freeman GJ (2002) The B7-CD28 superfamily. Nat Rev Immunol 2: 116–126. 11910893 10.1038/nri727

[20] Keir ME, Liang SC, Guleria I, Latchman YE, Qipo A, et al (2006) Tissue expression of PD-L1 mediates peripheral T cell tolerance. J Exp Med 203: 883–895. 16606670 10.1084/jem.20051776 PMC2118286

[21] Latchman Y, Wood CR, Chernova T, Chaudhary D, Borde M, et al (2001) PD-L2 is a second ligand for PD-1 and inhibits T cell activation. Nat Immunol 2: 261–268. 11224527 10.1038/85330

[22] Freeman GJ, Long AJ, Iwai Y, Bourque K, Chernova T, et al (2000) Engagement of the PD-1 immunoinhibitory receptor by a novel B7 family member leads to negative regulation of lymphocyte activation. J Exp Med 192: 1027–1034. 11015443 10.1084/jem.192.7.1027 PMC2193311

[23] Piconese S, Valzasina B, Colombo MP (2008) OX40 triggering blocks suppression by regulatory T cells and facilitates tumor rejection. J Exp Med 205: 825–839. 18362171 10.1084/jem.20071341 PMC2292222

[24] Takeda I, Ine S, Killeen N, Ndhlovu LC, Murata K, et al (2004) Distinct roles for the OX40-OX40 ligand interaction in regulatory and nonregulatory T cells. J Immunol 172: 3580–3589. 15004159 10.4049/jimmunol.172.6.3580

[25] Zheng G, Wang B, Chen A (2004) The 4-1BB costimulation augments the proliferation of CD4+ CD25+ regulatory T cells. J Immunol 173: 2428–2434. 15294956 10.4049/jimmunol.173.4.2428

[26] Ji HB, Liao G, Faubion WA, Abadia-Molina AC, Cozzo C, et al (2004) Cutting edge: the natural ligand for glucocorticoid-induced TNF receptor-related protein abrogates regulatory T cell suppression. J Immunol 172: 5823–5827. 15128759 10.4049/jimmunol.172.10.5823

[27] Wing K, Onishi Y, Prieto-Martin P, Yamaguchi T, Miyara M, et al (2008) CTLA-4 control over Foxp3+ regulatory T cell function. Science 322: 271–275. 18845758 10.1126/science.1160062

[28] Lam GK, Liao HX, Xue Y, Alam SM, Scearce RM, et al (2005) Expression of the CD7 ligand K-12 in human thymic epithelial cells: regulation by IFN-gamma. J Clin Immunol 25: 41–49. 15742156 10.1007/s10875-005-0356-5

[29] Cobbold SP, Castejon R, Adams E, Zelenika D, Graca L, et al (2004) Induction of foxP3+ regulatory T cells in the periphery of T cell receptor transgenic mice tolerized to transplants. J Immunol 172: 6003–6010. 15128783 10.4049/jimmunol.172.10.6003

[30] Stebbings R, Findlay L, Edwards C, Eastwood D, Bird C, et al (2007) “Cytokine storm” in the phase I trial of monoclonal antibody TGN1412: better understanding the causes to improve preclinical testing of immunotherapeutics. J Immunol 179: 3325–3331. 17709549 10.4049/jimmunol.179.5.3325

[31] Clamp M, Cuff J, Searle SM, Barton GJ (2004) The Jalview Java alignment editor. Bioinformatics 20: 426–427. 14960472 10.1093/bioinformatics/btg430

