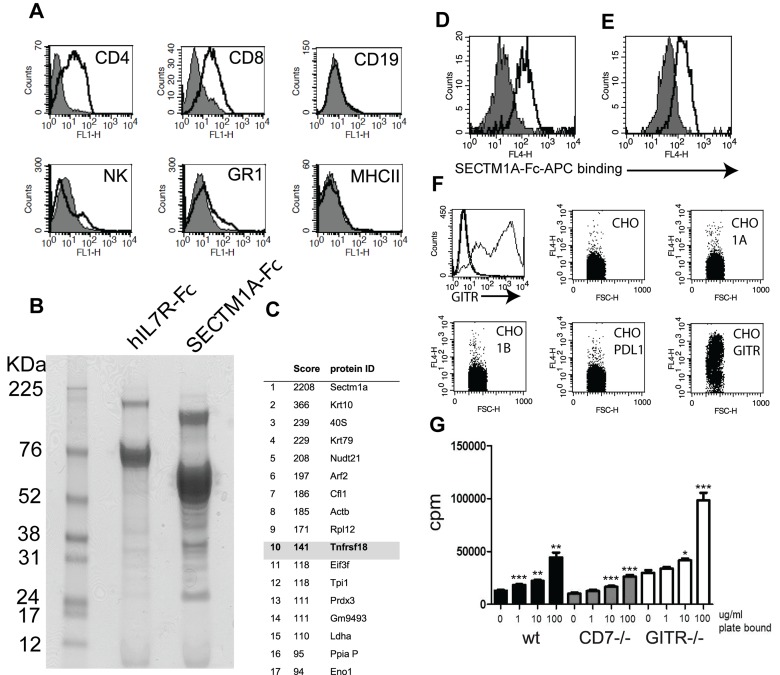# Correction: Secreted and Transmembrane 1A Is a Novel Co-Stimulatory Ligand

**DOI:** 10.1371/annotation/4d1054ad-beef-4fb2-9e53-815a58863fe2

**Published:** 2013-10-09

**Authors:** Duncan Howie, Hugo Garcia Rueda, Marion H. Brown, Herman Waldmann

As a result of errors in the production process, the version of Figure 6 that appeared in the article was incomplete. A correct version of the article is available here: 

**Figure:**